# The novel TERF2::PDGFRB fusion gene enhances tumorigenesis via PDGFRB/STAT5 signalling pathways and sensitivity to TKI in ph‐like ALL

**DOI:** 10.1111/jcmm.18114

**Published:** 2024-02-05

**Authors:** Guo‐fa Xu, Zhao Zeng, Zhi‐bo Zhang, Xiao‐mei Zhang, Man Wang, Qing Xiao, Jun Li, Xiao‐qing Xie, Sanxiu He, Hui‐hui Fu, Yi Liu, Zai‐liang Yang, Yu Chen, Jie Shi, Biao Wang, Hui‐ying Qiu, Qi Zhou, Yao Liu, Su‐ning Chen

**Affiliations:** ^1^ Department of Hematology Chongqing University FuLing Hospital, Chongqing, Central Laboratory, Chongqing University FuLing Hospital Chongqing China; ^2^ Jiangsu Institute of Hematology, National Clinical Research Center for Hematologic Diseases, Collaborative Innovation Center of Hematology, Institute of Blood and Marrow Transplantation The First Affiliated Hospital of Soochow University, Soochow University Suzhou China; ^3^ Department of Hematology‐Oncology Chongqing University Cancer Hospital Chongqing China; ^4^ Department of Hematology The Second Affiliated Hospital of Wannan Medical College Wuhu China; ^5^ Department of Hematology Affiliated Zhongshan Hospital of Dalian University Dalian China; ^6^ Department of Hematology The Third Affiliated Hospital of Soochow University (The First People's Hospital of Changzhou) Changzhou China

**Keywords:** CDX, Ph‐like acute lymphoblastic leukaemia, STAT5, TERF2::PDGFRB, TKI

## Abstract

Patients with Philadelphia chromosome‐like acute lymphoblastic leukaemia (Ph‐like ALL) often face a grim prognosis, with PDGFRB gene fusions being commonly detected in this subgroup. Our study has unveiled a newfound fusion gene, TERF2::PDGFRB, and we have found that patients carrying this fusion gene exhibit sensitivity to dasatinib. Ba/F3 cells harbouring the TERF2::PDGFRB fusion display IL‐3‐independent cell proliferation through activation of the p‐PDGFRB and p‐STAT5 signalling pathways. These cells exhibit reduced apoptosis and demonstrate sensitivity to imatinib in vitro. When transfused into mice, Ba/F3 cells with the TERF2::PDGFRB fusion gene induce tumorigenesis and a shortened lifespan in cell‐derived graft models, but this outcome can be improved with imatinib treatment. In summary, we have identified the novel TERF2::PDGFRB fusion gene, which exhibits oncogenic potential both in vitro and in vivo, making it a potential therapeutic target for tyrosine kinase inhibitors (TKIs).

## 1 INTRODUCTION

Acute lymphoblastic leukaemia (ALL) is a prevalent hematologic malignancy, with resistance or relapse serving as the primary cause of mortality. While childhood ALL boasts a cure rate exceeding 90%, the prognosis for adults with ALL is less favourable.^1^ Patients facing recurrence, regardless of age, generally experience a grim prognosis.^2^ As a result, there is a pressing need to pinpoint pivotal predictive and prognostic markers for ALL and formulate precise, targeted therapies to enhance clinical outcomes.

Philadelphia chromosome‐like acute lymphoblastic leukaemia (Ph‐like ALL), also known as BCR::ABL1‐like ALL, is characterized by a gene expression profile resembling that of ph + ALL but lacking the typical BCR::ABL1 fusion gene.^3^ Ph‐like ALL encompasses a wide spectrum of chromosomal rearrangements, mutations, and DNA copy number changes that disrupt cytokine receptor and tyrosine kinase (TK) signalling pathways.^4^ Several studies^5^, ^6^, ^7^ have consistently identified Ph‐like ALL as a high‐risk subgroup within B‐ALL. In recent years, Ph‐like ALL has garnered increasing attention and is now recognized as a distinct entity in the 5th edition of the World Health Organization Classification of Haematolymphoid Tumours: Lymphoid Neoplasms.^8^

The rearrangement of platelet‐derived growth factor receptor B (PDGFRBr) has been implicated in myeloproliferative neoplasms and has emerged as a target for tyrosine kinase inhibitors (TKI).^9^ Despite belonging to the ABL‐class of ph‐like B‐ALL, the fusion oncogene involving PDGFRB is rarely documented.^4^ A subset of B‐ALL cases with PDGFRBr rearrangements display resistance to conventional therapies.^10^ Hence, there is a pressing need to identify and investigate novel molecules associated with PDGFRBr to enhance the outcomes of patients with ph‐like ALL. In this study, we report the discovery of a novel TERF2::PDGFRB fusion gene in two individuals with ph‐like ALL.^11^ Remarkably, One patient experienced complete remission after receiving dasatinib in combination with chemotherapy. To comprehensively understand the role of TERF2::PDGFRB, we designed and conducted a series of experiments both in vitro and in vivo.

## 2 METHODS AND PATIENTS

We conducted an analysis of the genetic characteristics, clinical profiles and treatment outcomes in two cases of TERF2::PDGFRB‐positive Ph‐like ALL. The patients involved in our research provided informed consent, which was approved by the Ethics Committee of the First Affiliated Hospital of Soochow University. Our study was carried out in strict accordance with the principles outlined in the Declaration of Helsinki.

### 2.1 FISH (Fluorescence in situ hybridization, FISH)

FISH analysis was performed according to standard procedures with locus‐specific probes spanning the PDGFRB locus (Abbott Diagnostics, Wiesbaden, Germany). At least 200 cells were analysed.

### 2.2 RNA extraction, cDNA synthesis and PCR

RNA was extracted using TrizolTM reagent (Invitrogen, Karlsruhe, Germany).

RNA reverse transcription was done using 5 × PrimeScript RT Master Mix (TaKaRa).

The primers used for the TERF2::PDGFRB gene were as followed: (TERF2 forward: 5′‐ AGCATCACCAGCCCTCAAAA ‐ 3′); (PDGFRB reverse: 5′‐ CTCCTCCTCCCAGTACGTCA ‐ 3′).

Cycled with the following conditions: 95 C × 3 min (1 cycle), 95 C × 15 s, 55 C × 20s and 72 C × 50 s (35 cycles), 72 C × 7 min (1 cycle) and 4 C hold.

### 2.3 Cell culture condition

Ba/F3 cells were maintained at a density between (0.5–2) × 10^6^ in RPMI/1640 (Gibcol) with 10% fetal bovine serum (FBS; HyClone), penicillin–streptomycin and PeproTech recombinant (m) mIL‐3 at 10 ng/mL each.

Hela cells and 293 T cells were grown in DMEM medium with 10% FBS (HyClone) and penicillin–streptomycin.

All cells were grown at 37°C in 5% CO_2_.

### 2.4 Generation of FLAG and GFP‐tagged constructs

The plasmids were synthesized by Nanjing Qingke Biotechnology Co., LTD. Lentivirus overexpression vector [MED14‐HOXA9 in Venus (Ampicillin)] was tagged the Flag at the N terminal, venus produced from an independent ORF with an IRES(pCAG‐MCS‐IRES‐Venus). Because venus itself has yellow fluorescence, Flag and YFP are not marked on venus. GFP and YFP are the same fluorescent channel in flow cytometry, so they are collectively referred to as GFP in this paper. pCDNA3 plasmids (TERF2, PDGFRB and TERF2::PDGFRB) only were labelled Flag and did not labelled any fluorescence. Details of the plasmids are shown in Appendix S1.

### 2.5 Transfection of Ba/F3 and Hela cells

Using lentivirus, we expressed TERF2::PDGFRB proteins in Ba/F3 cells cultured with interleukin‐3 (IL‐3). On day 3 post‐infection, we purified GFP+ cells.

Lentivirus for 293 t transfections was obtained by adding 1.56 μg VSVG +2.89ug △R + 1.11 μg REV +4.44ug TERF2::PDGFRB plasmids together for 10 min. Over the next 6 h, 10% FBS was added to the media and was changed daily. Viral supernatants were collected after 24 and 48 h, pooled, and concentrated 5× with Retro Concentin (System Biosciences) following manufacturer instructions. For Ba/F3 cells, concentrated viral supernatant was bound to RetroNectin (Takara Bio, Otsu Shiga, Japan) coated 6‐well plates following manufacturer instructions. Ba/F3 cells were transduced by spining at 1800 RPM and 32°C for 45 min and then cultured as described.

Ba/F3 cells were infected by mixing 1 × 10^6^ cells with 1 mL of 5× concentrated virus in a 6‐well dish. The following day, cells were washed and grown in fresh media.

Hela cells transfection was done by adding 200 μL jet Prime@buffer +2ug TERF2::PDGFRB plasmids + jet Prime 4 μL together for 10 min.

### 2.6 Immunofluorescence staining

Hela cells carrying TERF2::PDGFRB were fixed with 4% paraformaldehyde solution for 10 min on ice, permeabilized with 0.25% Triton X‐100 for 10 min at room temperature, washed twice with PBS and blocked with 1% BSA for 2 h at room temperature. The samples were then incubated with anti‐Flag antibody (Sigma) diluted 1:100 in 1% BSA overnight at room temperature. Primary antibodies were detected using a secondary antibody (rabbit anti‐mouse Alexa Fluor plus 649) diluted 1:200 with 1% BSA for 2 h at room temperature. The samples were then washed twice. DAPI (5 μg/mL) was added, the samples were examined under an Axio Imager Z2 fluorescence microscope (ZEISS), and images were taken at 100× magnification using a Zeiss LSM 700 confocal fluorescence microscope and Zen imaging software (Carl Zeiss Microscopy, Switzerland).

Mouse tissues were fixed in 4% paraformaldehyde solution, mounted in frozen blocks, sliced into 4 μm sections, and stained with haematoxylin and eosin.

### 2.7 Proliferation assay

For proliferation assays, 2 × 10^3^ or 5 × 10^3^ cells per well were seeded in triplicates in the presence or absence of IL‐3 in a 96‐well dish. Cells were incubated and quantified at 24 h intervals for 72 h using the CCK8 (Promega, Madison, WI) recording absorbance at 490 nm with a 96‐well plate reader.

### 2.8 Immunoblotting

Ba/F3 cells with empty vector or TERF2::PDGFRB were washed twice with ice‐cold PBS and then lysed using lysis buffer. Total cell lysate (20 μg) was heated at 95°C for 5 min in the sample buffer, subjected to PAGE, and then electrotransferred onto nitrocellulose membranes. Detection was performed using HRP‐conjugated secondary antibodies and enhanced chemiluminescence. Stat5 (#94205), phospho‐Stat5 (Tyr694) (#9314) and actin antibodies were purchased from Cell Signaling Technology.

### 2.9 In vitro cytokine‐independent growth assay in Ba/F3 cells

Ba/F3 cells were transduced with venus and TERF2::PDGFRB, sorted on GFP+ populations, washed and rested for 24 h, after which 1,000,000 cells were expanded in the absence of IL‐3. Cells were maintained at a density of 0.2 × 10^6^–2 × 10^6^ per mL. Cells were enumerated every other day with a haemocytometer.

### 2.10 FACS analysis of Ba/F3 apoptosis

One million cells per well were seeded in triplicate in the absence of IL‐3 in a 6‐well dish. After 48 h, cells were incubated with 100 μL binding buffer+ Annexin V (United Biology, China) 5 μL +7‐AAD (United Biology, China) 5 μL for 15 min in the dark.

### 2.11 Tyrosine kinase inhibitor (imatinib) cytotoxicity

Ba/F3 cells overexpressing the TERF2::PDGFRB fusion gene were incubated with increasing concentrations of imatinib (0 nM, 1 nM, 10 nM, 100 nM, 1uM, 10μM and 100μM) and dasatinib (0 nM, 0.001 nM, 0.010 nM, 0.100 nM, 1 nM, 10 nM and 100 nM) for 48 h; after which cell viability was measured using CCK8 assay. Experiments were performed in triplicate and repeated for at least three times. The plots were drawn using Graph Prism 5.

### 2.12 In vivo leukemogenesis

After sublethal irradiation, 300,000 Ba/F3 cells were transplanted by tail vein injection into 6–8‐week‐old wild‐type Balb/c mice (5 Gy). There were five mice in each group. Mice were kept in SPSS barriers. Animals were monitored and sacrificed during moribund or clinical manifestations of the central nervous system. Post‐mortem flow cytometry of bone marrow and spleen was done for GFP+. Unexpected death without relevance to leukaemia was considered an exclusion criterion for survival curves.

## 3 RESULTS

### 3.1 Cases presentation

#### 3.1.1 Cases 1

**FIGURE 1:**
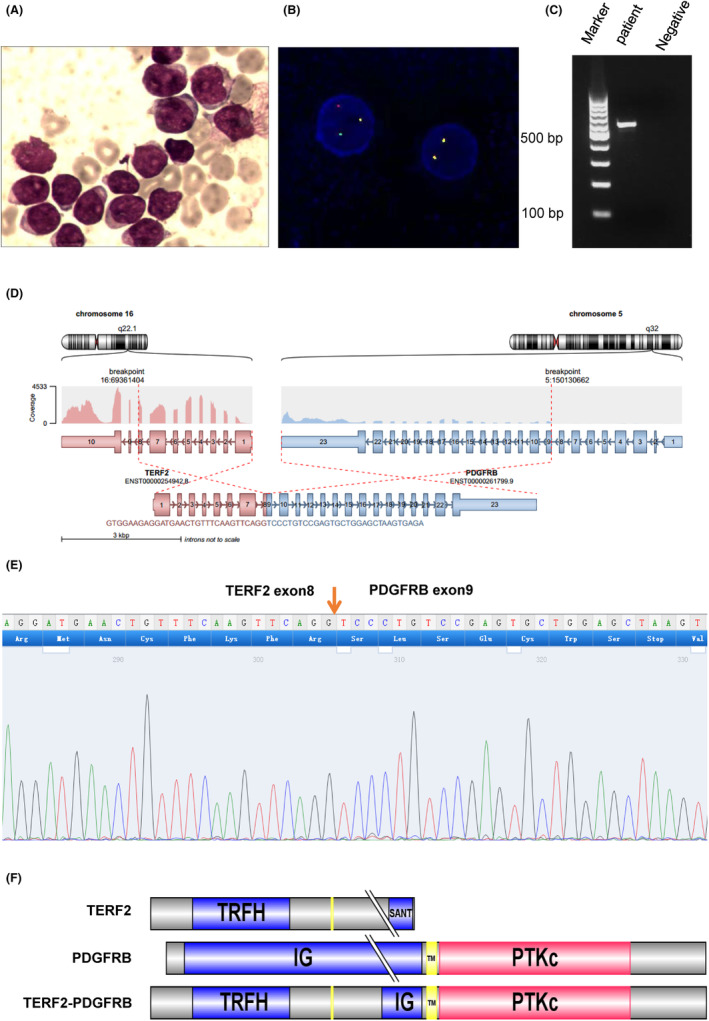
The information of patient and structure of the TERF2::PDGFRB fusion gene: (A) Ninety percent blasts in the bone marrow. (B) Yellow fluorescence illustrates PDGFRB rearrangement as observed in FISH. (C) The expression of the fusion gene was confirmed through RT‐PCR in patient. (D–F) Displays the fusion of exon 9–23 of PDGFRB with exon 1–8 of the TERF2 gene.

In June 2020, a 26‐year‐old woman received a diagnosis of B‐cell acute lymphoblastic leukaemia (ALL) characterized by leukocytosis (124.79 × 10^9^/L), severe anaemia (51 g/L) and a low platelet count (55 × 10^9^/L). A bone marrow aspiration revealed the presence of 90% blasts (Figure 1A). Phenotypically, these cells exhibited positivity for CD10, CD19, CD20, CD34, CD33 and cCD79a, consistent with a B‐ALL diagnosis. Cytogenetic analysis using fluorescence in situ hybridization (FISH) indicated a PDGFRB rearrangement (Figure 1B). Furthermore, targeted RNA sequencing (RNA‐seq) of leukaemia blast cells revealed the presence of a novel TERF2::PDGFRB fusion gene, involving an in‐frame fusion of exon 9–23 of PDGFRB with exon 1–8 of the TERF2 gene (Figure 1C–F).

The patient initially did not respond to conventional chemotherapy (CIVP + EA: cyclophosphamide 0.3 g D1‐3, vindesine 4 mg D4,11,18, idarubicin 10 mg D4,11, dexamethasone 10 mg D4‐10, 7.5 mg D11‐17, 5 mg D18‐24, 2.5 mg D25‐31, Cytarabine 150 mg D16‐20, etoposide 100 mg D16‐17) and failed to achieve complete remission (At day 14, blast cell and minimal residual disease counted for 76.5% and 91.7%, respectively, in bone marrow). At day 22, targeted RNA‐seq revealed the presence of a novel TERF2::PDGFRB fusion gene. Subsequently, she received treatment with dasatinib (100 mg qd) in combination with chemotherapy (Continuation of the dexamethasone in the CIVP programme), resulting in a significant reduction in blast percentage from 91.7% to 15.1% within 14 days and further down to 0.52% within 41 days. The detailed medication regimen for the first course of treatment was shown in Table S1. Real‐time quantitative polymerase chain reaction (qRT‐PCR) demonstrated the absence of the TERF2::PDGFRB fusion gene after 4 months of treatment with dasatinib, followed by treatment with autologous CD19 CAR‐T cells. Subsequently, she underwent haploid haematopoietic stem cell transplantation. As of 13 September 2023, she has remained free of disease for 33 months post‐transplant.

#### 3.1.2 Cases 2

Through RNA sequencing of 170 B‐ALL patients, we identified another individual harbouring the TERF2::PDGFRB fusion gene. This patient exhibited the same fusion sites as the previously mentioned case but lacked the Ph + and MLL rearrangement, as well as the E2A/PBX1 fusion.

The patient, a 45‐year‐old man, received a diagnosis of B‐ALL in September 2015 based on an abnormal complete blood count (WBC 5.15*10^9^/L, Hb 70 g/L, PLT 14*10^9^/L). Lymphoblasts were observed in both the peripheral blood and the bone marrow, accounting for 74% of the cellular composition in the bone marrow. Following treatment with the IVP regimen (Idarubicin+vindesine+dexamethasone), the minimal residual disease (MRD) levels were measured at 17.8% on day 14 and 23% on day 33 using FMC. Subsequent chemotherapy with the MOLP regimen (Mitoxantrone + Vindesine + P‐ASP + Dexamethasone) led to a complete response in the bone marrow; however, MRD remained detectable. Unfortunately, the patient did not return for follow‐up after discharge.

### 3.2 Subcellular mislocalization of TERF2::PDGFRB

**FIGURE 2:**
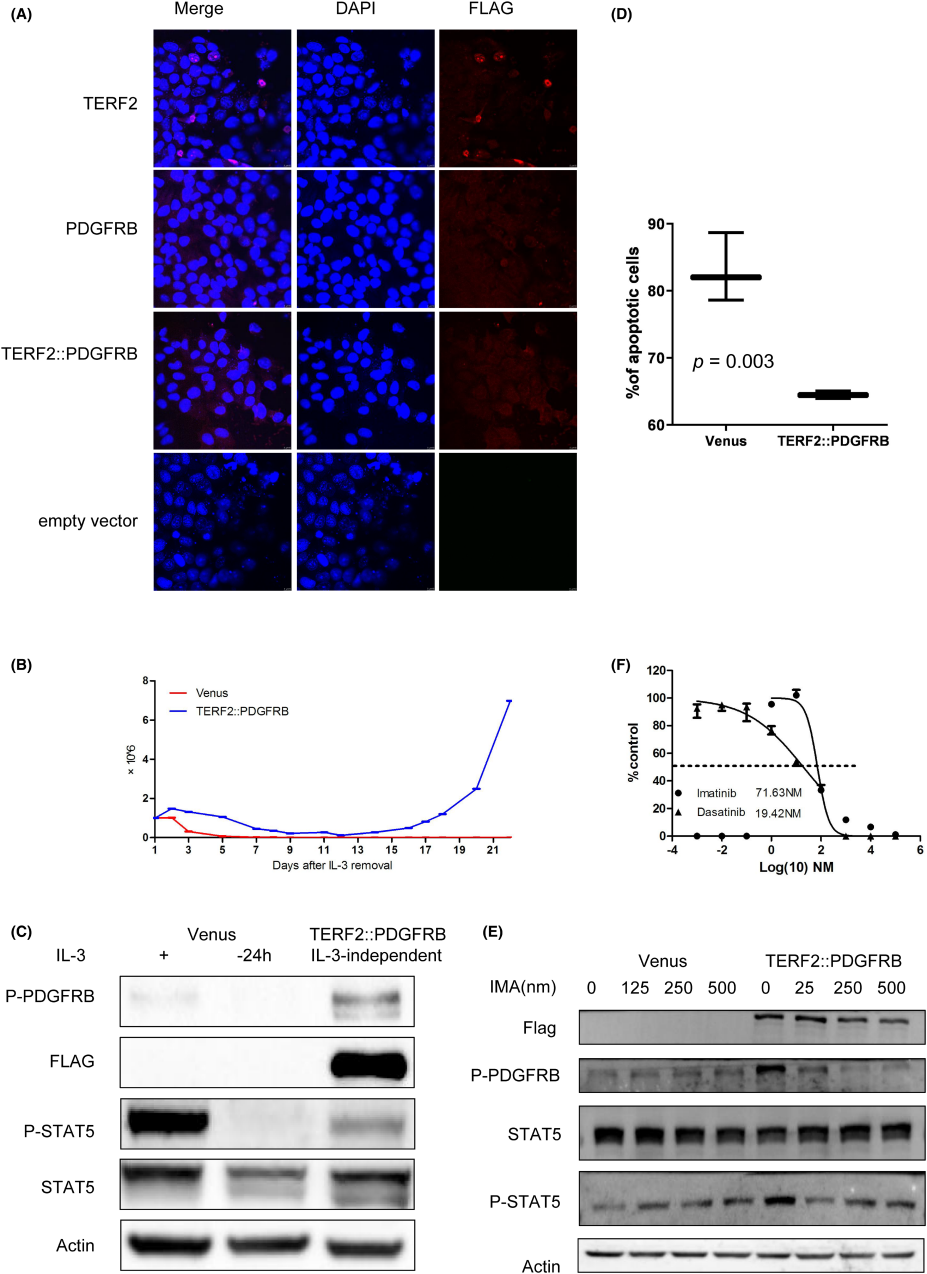
Functional studies of the TERF2::PDGFRB fusion gene: (A) Depicts the subcellular mislocalization of TERF2::PDGFRB. (B) After 12 days, cells bearing the TERF2::PDGFRB fusion gene demonstrated IL‐3‐independent cell proliferation. (C) Show how the TERF2::PDGFRB fusion gene induces IL‐3‐independent growth through pPDGFRB and pSTAT5. (D) Demonstrates the apoptosis rate of Ba/F3 cells transduced with the TERF2::PDGFRB fusion gene compared to Venus after 48 h of IL‐3 deprivation. (E) Display the sensitivity of the TERF2::PDGFRB fusion gene to imatinib and the reversal of PDGFRB and STAT5 activation by imatinib. (F) Imatinib and dasatinib effectively inhibited the proliferation of Ba/F3 cells, with IC50 of 71.63 nM and 19.42 nM.

The TERF2, PDGFRB and TERF2::PDGFRB fusion genes were overexpressed in Hela cells. Confocal imaging of Hela cells showed that the TERF2 and PDGFRB genes were situated in the nucleus and cytoplasm/plasma membrane, respectively. By contrast, TERF2::PDGFRB was primarily localized in the plasma membrane and cytoplasm, with minimal detection within the nuclei, as observed in immunofluorescence staining (Figure 2A).

### 3.3 TERF2::PDGFRB fusion gene induces IL‐3‐independent growth through pPDGFRB and pSTAT5

A plasmid containing the TERF2::PDGFRB fusion gene was constructed and introduced into the murine Ba/F3 cell line using a lentiviral vector to assess the proliferative activity associated with this fusion gene. The expression of the fusion gene was confirmed through RT‐PCR (Figure S1A). Furthermore, fusion protein expression was validated via Western blot analysis. As anticipated, cells harbouring the TERF2::PDGFRB fusion gene exhibited significantly higher proliferation in the absence of IL3 compared to cells containing an empty vector (Figure S1B). Notably, cells bearing the TERF2::PDGFRB fusion gene demonstrated IL‐3‐independent cell proliferation (Figure 2B) along with the activation of p‐PDGFRB and Stat5 signalling pathways (Figure 2C).

### 3.4 Apoptosis inhibition and cell cycle alteration induced by TERF2::PDGFRB fusion gene

Following 48 h of IL‐3 deprivation, the apoptosis rate of Ba/F3 cells transduced with TERF2::PDGFRB was markedly lower compared to those carrying an empty vector, as illustrated in Figure 2D. Additionally, there were no significant alterations observed in the cell cycle of Ba/F3 cells with the TERF2::PDGFRB fusion gene, whether exposed to IL‐3 or not, as shown in Figure S1C,D.

### 3.5 The TERF2::PDGFRB fusion gene was sensitive to imatinib

Our study examined the impact of imatinib on the proliferation of Ba/F3 cells bearing the PDGFRB fusion gene in the absence of IL3 expression. Moreover, imatinib demonstrated a dose‐dependent reversal of PDGFRB and STAT5 activation in TERF2::PDGFRB‐positive Ba/F3 cells (Figure 2E). Imatinib and dasatinib effectively inhibited the proliferation of Ba/F3 cells, with IC50 of 71.63 nM and 19.42 nM (Figure 2F).

### 3.6 Tumorigenicity of TERF2::PDGFRB fusion gene in vivo

Ectopic expression of the TERF2::PDGFRB fusion gene effectively induced IL3‐independent growth of Ba/F3 cells, confirming its oncogenic potential in vitro. To further assess the tumorigenicity of the TERF2::PDGFRB fusion gene in vivo, we initiated sublethal irradiation in BALB/c mice and subsequently transplanted 3 × 10^5^ Ba/F3 cells carrying TERF2::PDGFRB or an empty vector via tail vein injection. The mice were categorized into three groups: empty vector (BV), experimental groups carrying the TERF2::PDGFRB fusion gene without imatinib (BT‐IMA), and with imatinib treatment (BT + IMA). Starting from the 10th day after transplantation, the BT + IMA group received imatinib (100 mg/kg.d) continuously by gavage for 14 days, while the BV and BT‐IMA groups were given an equivalent volume of PBS.

**FIGURE 3:**
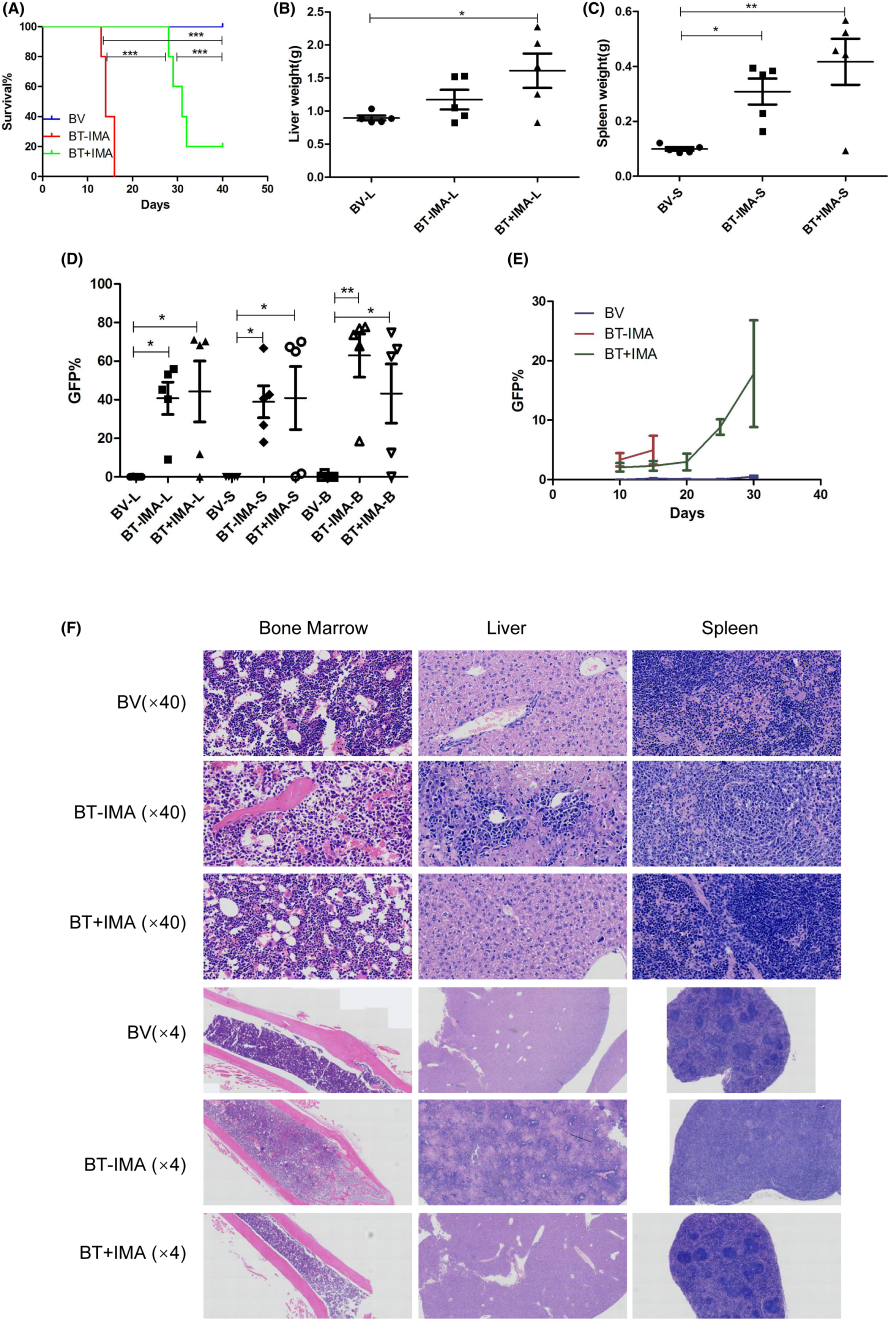
Tumorigenicity of the TERF2::PDGFRB fusion gene in vivo: (A) Depicts the survival comparison between TERF2::PDGFRB mice and empty vector controls. The survival of TERF2::PDGFRB mice significantly decreased in comparison to age‐matched empty vector controls. Mice expressing the TERF2::PDGFRB fusion gene displayed sensitivity to imatinib, resulting in significantly prolonged survival in BT + IMA mice than BT‐IMA mice. (B) Shows the average liver weight of the mice. The average liver weight of BT + IMA mice was significantly greater than that of empty vector controls. (C) Demonstrates the average spleen weight of the mice. The average spleen weight of both BT‐IMA mice and BT + IMA mice was significantly higher than that of empty vector controls. (D) Displays the GFP levels in the liver, spleen and bone marrow of the mice. Flow cytometry analysis demonstrated a substantial increase in GFP expression in the liver, spleen and bone marrow of moribund or deceased mice bearing the TERF2::PDGFRB fusion gene. (E) Illustrates the GFP levels in peripheral blood after the withdrawal of imatinib. After discontinuation of imatinib, peripheral blood GFP levels gradually increased. (F) Provides histological examination results of the bone, liver and spleen. Histological examination revealed extensive infiltration of TERF2::PDGFRB‐positive leukaemia cells into various tissues, including bone, liver and spleen.

The TERF2::PDGFRB gene fusion resulted in fully penetrant leukaemia development. The survival of TERF2::PDGFRB mice significantly decreased in comparison to age‐matched empty vector controls (*p* < 0.001, Figure 3A). The average liver weight of BT + IMA mice was significantly greater than that of empty vector controls (1.612 ± 0.583 vs. 0.895 ± 0.08 g, *p* < 0.05) (Figure 3B). Moreover, the average spleen weight of both BT‐IMA mice (0.308 ± 0.105 g, *p* < 0.05) and BT + IMA mice (0.417 ± 0.188 g, *p* < 0.01) was significantly higher than that of mice in the control group (0.099 ± 0.015 g) (Figure 3C). Flow cytometry analysis demonstrated a substantial increase in GFP expression in the liver, spleen and bone marrow of moribund or deceased mice bearing the TERF2::PDGFRB fusion gene (Figure 3D). Histological examination revealed extensive infiltration of TERF2::PDGFRB‐positive leukaemia cells into various tissues, including bone, liver and spleen (Figure 3F).

We evaluated the effects of the TERF2::PDGFRB fusion gene on drug sensitivity in vivo. Mice expressing the TERF2::PDGFRB fusion gene displayed sensitivity to imatinib, resulting in significantly prolonged survival (*p* < 0.001), with a median survival of 31 (28‐) days in BT + IMA mice and 14 (13–16) days in BT‐IMA mice (*p* < 0.001, Figure 3A). However, after discontinuation of imatinib, peripheral blood GFP levels gradually increased, ultimately leading to the demise of the mice (Figure 3A,E).

## 4 DISCUSSION

Harvey RC et al. reported that PDGFRB rearrangement was identified in 1.6% of the 2506 cases with BCR‐ABL1‐like ALL.^12^ Up to now, only a minority of fusion partners of PDGFRB, most commonly ETV6‐PDGFRB and EBF1‐PDGFRB, have been recurrently reported, while the vast majority have only been described in case reports.^13^, ^14^, ^15^, ^16^, ^17^ PDGFRB rearrangement remains a rare and less‐studied subtype of Ph‐like ALL. Therefore, there is a clear need for both in vitro and in vivo investigations of the TERF2::PDGFRB fusion gene. Our study represents the first report of Ph‐like ALL with a recurring TERF2::PDGFRB fusion. Furthermore, we have demonstrated that TERF2::PDGFRB‐dependent leukemogenesis hinges on dysregulated tyrosine kinase activity. Additionally, we have employed a genetic mouse model to confirm that the TERF2::PDGFRB fusion gene is tumorigenic in vivo and responsive to imatinib treatment.

Previous studies have reported that the breakpoints of PDGFRB rearrangement are often found at exon 11 or 12, such as ETV6::PDGFRB, WNK1::PDGFRB, TNIP1::PDGFRB, PCM1::DGFRB, CD74::DGFRB, ATF7IP::PDGFRB, AGGF1::PDGFRB (at exon 11), DOCK2::PDGFRB, SATB1::PDGFRB, SART3::PDGFRB and G3BP1::PDGFRB (at exon 12).^18^ The general structure of a fusion receptor consists of an N‐terminal domain derived from a partner protein, and the C‐terminal part of the receptor. The expression of these chimeric oncogenes is always controlled by the promoter of the partner gene, allowing the activation of receptors by constitutively phosphorylated. The paradigm for constitutive activation of fusion receptors is enforced dimerization mediated by specific domains present in the N‐terminal portion of the partner protein.^19^ Most of the fusion partners identified contain self‐association domains such as a pointed domain or coiled‐coil repeats. For example, the constitutive activation of ETV6::PDGFRB is due to enforced homodimerization by self‐association domains present in the fusion partner protein (ETV6), which in turn activates downstream signalling pathways resulting in cell proliferation and survival.^20^ The product from the CD74^intr^::PDGFRB fusion [a fusion between CD74 (exon 1) and PDGFRB (exon 11)] likely arises from an internal ATG within PDGFRB itself, encoding a truncated PDGFRB molecule.^21^

However, the breakpoint in TERF2::PDGFRB is unusual (exon 9), although it was already described in one case of ETV6::PDGFRB and KANK1::PDGFRB. Because the TERF2::PDGFRB fusion gene includes the transmembrane region of the PDGFRB gene, the fusion protein is localized in the membrane and cytoplasm as shown in Figure 2A. TERF2 contains TRF homology (TRFH) dimerization domain (α helix structure). As stated in the previous paragraph, the high probability of activation of TERF2::PDGFRB may be due to be controlled by the promoter of TERF2, allowing the activation of receptors by constitutively phosphorylated. Could the loss of one TERF2 allele affect telomere maintenance? The TERF2::PDGFRB fusion gene lost the C‐terminal domain of TERF2 which is mainly responsible for the specific recognition and binding of the telobox DNA repeat sequence, TTAGGG. This change is likely to affect telomere maintenance. However, Markiewicz‐Potoczny M and colleagues find that TRF2 is dispensable for the proliferation and survival of mouse embryonic stem (ES) cells.^22^ Further experiments are needed to demonstrate the effect of TERF2::PDGFRB fusion gene on telomere maintenance.

Consistent with our findings, some studies have reported that PDGFRB rearrangement leads to increased proliferation and reduced apoptosis of Ba/F3 cells.^15^, ^16^ Kathryn G. Roberts et al.^23^ demonstrated that treatment with dasatinib as a single agent resulted in a cytostatic effect in leukaemia cells harbouring the EBF1‐PDGFRB fusion. Our study further verifies the sensitivity of the TERF2::PDGFRB fusion gene to imatinib in animal models. Patients with PDGFRB fusion‐positive ALL may benefit from the addition of TKIs to chemotherapy. Despite some ongoing trials (NCT03564470, NCT02883049, ChiCTR2000029475 and NCT02143414), controlled studies in this area are scarce, and evidence supporting TKI use in this subgroup is primarily derived from case reports.^14^, ^24^, ^25^, ^26^, ^27^, ^28^ In our case, the patient achieved a rapid and complete remission following the addition of dasatinib. However, achieving MRD negativity was not accomplished with dasatinib alone. Consequently, for high‐risk patients, after achieving complete remission with dasatinib, consideration should be given to treatments such as CAR‐T cell therapy or allo‐HSCT.

In our study, we have identified and functionally characterized the novel TERF2::PDGFRB fusion gene in Ph‐like ALL. Our results underscore the significant role of PDGFRB in the pathogenesis of Ph‐like ALL. The favourable response to dasatinib underscores the importance of accurate diagnosis and targeted therapy in managing this condition.

## 5 CONCLUSIONS

Ph‐like ALL with the TERF2::PDGFRB fusion gene has demonstrated resistance to conventional induction chemotherapy regimens, making it challenging to achieveMRD negativity. However, the patients in our study exhibited a positive response to dasatinib treatment. Our findings suggest that the TERF2::PDGFRB fusion gene promotes the growth of Ba/F3 cells independently of IL‐3, leading to the activation of the p‐PDGFRB and p‐STAT5 signalling pathways, which can be effectively inhibited by imatinib. Moreover, our experiments using BALB/c mice have shown that Ba/F3 cells carrying the TERF2::PDGFRB fusion gene are capable of inducing leukaemia in vivo and are notably sensitive to imatinib treatment.

## AUTHOR CONTRIBUTIONS

**Guo‐fa XU:** Writing – original draft (equal). **Zhao Zeng:** Methodology (equal). **Zhi‐bo Zhang:** Methodology (equal). **Xiao‐mei Zhang:** Conceptualization (supporting). **Man Wang:** Investigation (supporting). **Qing Xiao:** Validation (supporting). **Jun Li:** Visualization (supporting). **Xiao‐qing Xie:** Supervision (supporting). **Sanxiu He:** Software (supporting). **Hui‐hui Fu:** Visualization (supporting). **Yi Liu:** Data curation (supporting). **Zai‐liang Yang:** Conceptualization (supporting). **Yu Chen:** Software (supporting). **Jie Shi:** Formal analysis (supporting). **Biao Wang:** Resources (supporting). **Hui‐ying Qiu:** Funding acquisition (supporting). **Qi Zhou:** Writing – review and editing (equal). **Yao Liu:** Writing – review and editing (equal). **Su‐ning Chen:** Writing – original draft (lead).

## FUNDING INFORMATION

This work was supported by Postdoctoral Science Foundation of Chongqing Natural Science Foundation, No.CSTB2023NSCQ‐BHX0232, Fuling District Science and Technology Project of Chongqing (FLKJ, 2021ABB1025). Health Development Promotion Project—a public welfare project to help national public hospitals improve their scientific research capacity (KM‐ZLGJ‐049).

## CONFLICT OF INTEREST STATEMENT

The authors declare no competing financial interests.

## Supporting information


Appendix S1

Figure S1

Table S1

## Data Availability

The data that support the findings of this study are available from the corresponding author upon reasonable request.
