# Supplementary figures and images for: The novel TERF2::PDGFRB fusion gene enhances tumorigenesis via PDGFRB/STAT5 signalling pathways and sensitivity to TKI in ph‐like ALL
Source: J Cell Mol Med. 2024 Feb 5;28(3):e18114. doi: 10.1111/jcmm.18114 (PMC10844707; doi:10.1111/jcmm.18114)

MED14-HOXA9 in Venus (Ampicillin)


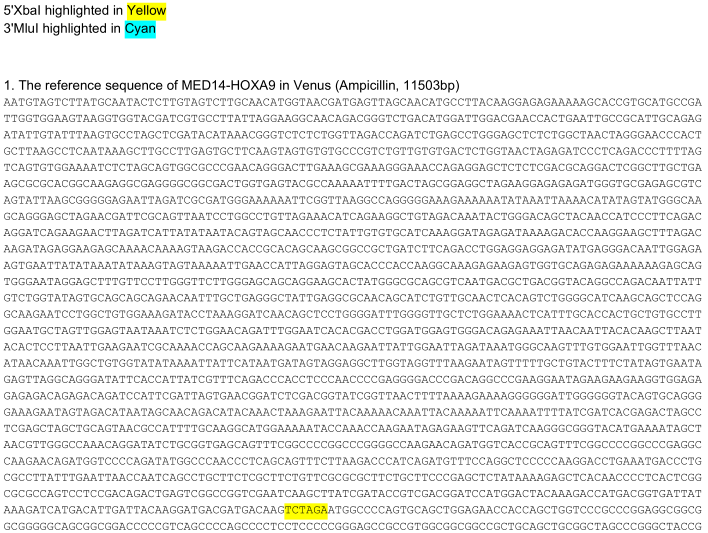


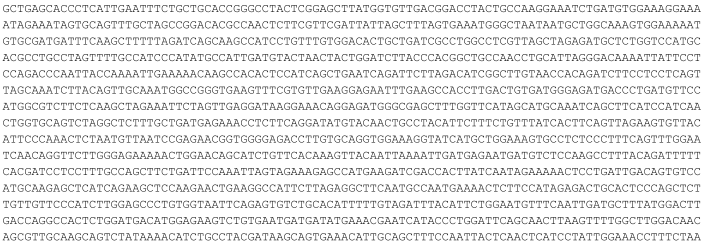


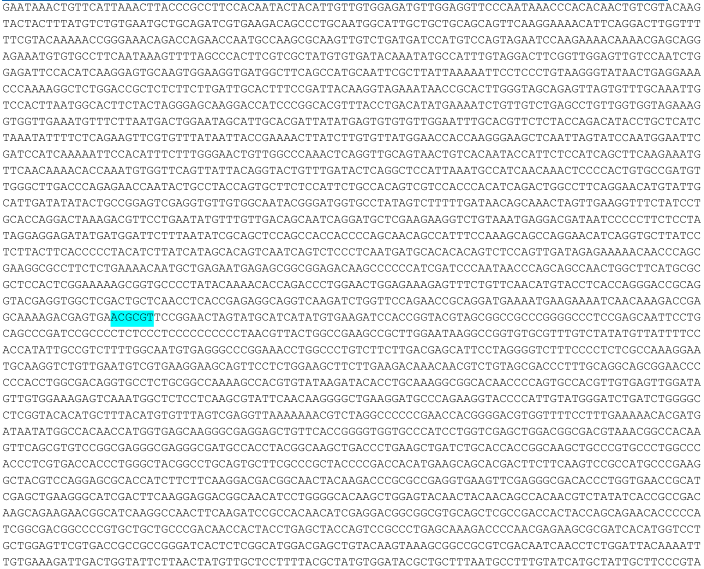


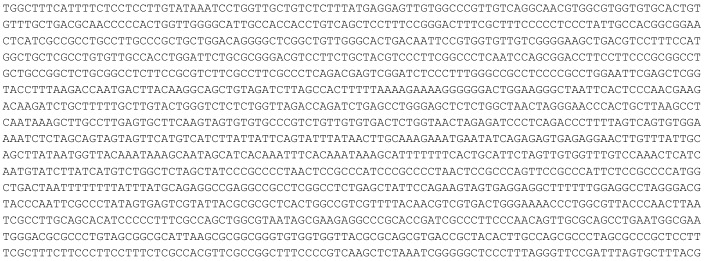


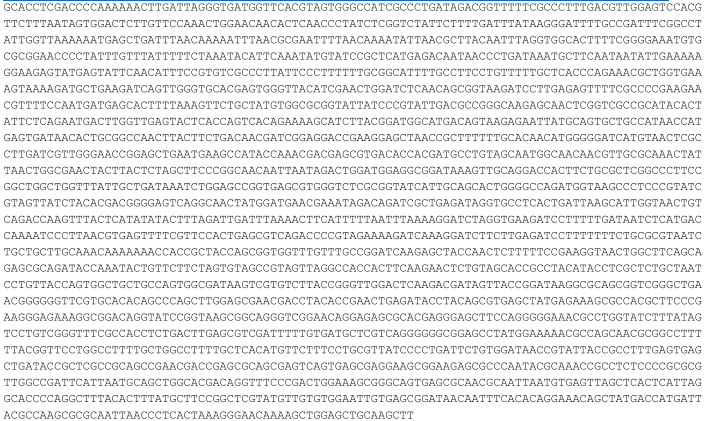

Supplement: Supplementary file 1 — Appendix S1 [file JCMM-28-e18114-s003.zip › jcmm18114-sup-0003-Supinfo03.docx]

A

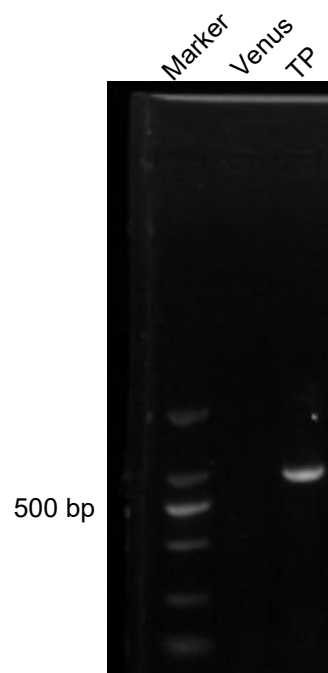

B

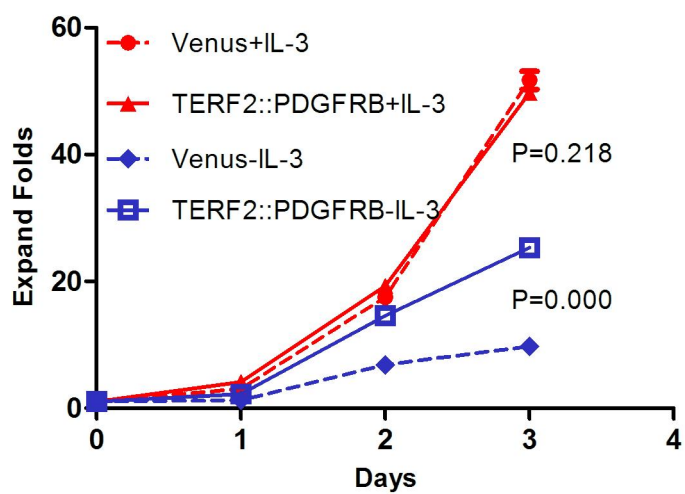

C

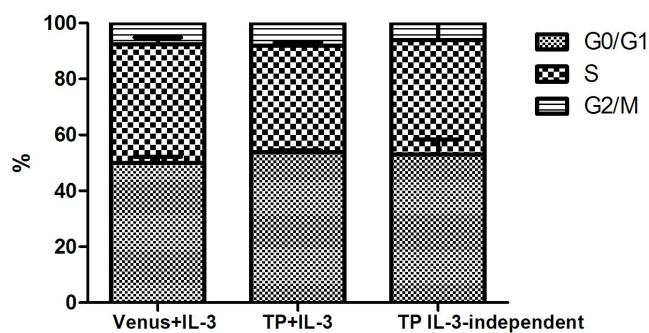

D

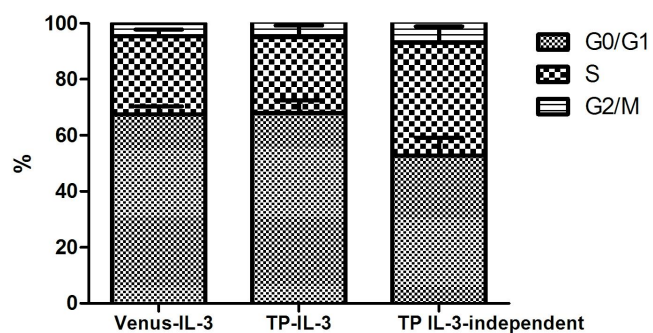

Supplement: Supplementary file 2 — Figure S1 [file JCMM-28-e18114-s001.zip › jcmm18114-sup-0002-FigureS1.pdf]
