# Supplementary material for: The novel TERF2::PDGFRB fusion gene enhances tumorigenesis via PDGFRB/STAT5 signalling pathways and sensitivity to TKI in ph‐like ALL
Source: J Cell Mol Med. 2024 Feb 5;28(3):e18114. doi: 10.1111/jcmm.18114 (PMC10844707; doi:10.1111/jcmm.18114)
Supplement: Supplementary file 2 — Figure S1 [file JCMM-28-e18114-s001.zip › Figure S1 caption.docx]

Figure S1 Validation, proliferation and cell cycle of the TERF2::PDGFRB fusion gene: (A) The expression of the fusion gene was confirmed through RT-PCR. The TERF2::PDGFRB fusion gene was successfully transferred into Ba/F3 cells. (B) The TERF2::PDGFRB fusion gene had a proliferation advantage compared with empty vector in the medium without IL-3. (C and D) There was no significant change in cell cycle in Ba/F3 cells with TERF2::PDGFRB and venus with(C) or without IL-3(D).
