# Supplementary material for: The novel TERF2::PDGFRB fusion gene enhances tumorigenesis via PDGFRB/STAT5 signalling pathways and sensitivity to TKI in ph‐like ALL
Source: J Cell Mol Med. 2024 Feb 5;28(3):e18114. doi: 10.1111/jcmm.18114 (PMC10844707; doi:10.1111/jcmm.18114)
Supplement: Supplementary file 3 — Table S1 [file JCMM-28-e18114-s002.docx]

Supplementary table 1 The details of drugs for patient one

| Date | Day | CTX  (mg) | Vindesine  (mg) | IDA  (mg) | DEX  (mg) | Ara-c  (mg) | VP-16  (mg) | Dasatinib  (mg) | Others |
| --- | --- | --- | --- | --- | --- | --- | --- | --- | --- |
| 6-26-2020 | 1 | 300 |  |  |  |  |  |  |  |
| 6-27 | 2 | 300 |  |  |  |  |  |  |  |
| 6-28 | 3 | 300 |  |  |  |  |  |  |  |
| 6-29 | 4 |  | 4 | 10 | 10 |  |  |  |  |
| 6-30 | 5 |  |  |  | 10 |  |  |  |  |
| 7-1 |  |  |  |  | 10 |  |  |  |  |
| 7-2 | 7 |  |  |  | 10 |  |  |  |  |
| 7-3 | 8 |  |  |  | 10 |  |  |  |  |
| 7-4 | 9 |  |  |  | 10 |  |  |  |  |
| 7-5 | 10 |  |  |  | 10 |  |  |  |  |
| 7-6 | 11 |  | 4 | 10 | 7.5 |  |  |  |  |
| 7-7 | 12 |  |  |  | 7.5 |  |  |  |  |
| 7-8 | 13 |  |  |  | 7.5 |  |  |  |  |
| 7-9 | 14 |  |  |  | 7.5 |  |  |  | Blast cell: 76.5%,  MRD：91.7% |
| 7-10 | 15 |  |  |  | 7.5 |  |  |  |  |
| 7-11 | 16 |  |  |  | 7.5 | 150 | 100 |  |  |
| 7-12 | 17 |  |  |  | 7.5 | 150 | 100 |  |  |
| 7-13 | 18 |  | 4 |  | 5 | 150 |  |  |  |
| 7-14 | 19 |  |  |  | 5 | 150 |  |  |  |
| 7-15 | 20 |  |  |  | 5 | 150 |  |  |  |
| 7-16 | 21 |  |  |  | 5 |  |  |  |  |
| 7-17 | 22 |  |  |  | 5 |  |  | 100 | RNA-seq:TERF2-PDGFRB |
| 7-18 | 23 |  |  |  | 5 |  |  | 100 |  |
| 7-19 | 24 |  |  |  | 5 |  |  | 100 |  |
| 7-20 | 25 |  |  |  | 2.5 |  |  | 100 |  |
| 7-21 | 26 |  |  |  | 2.5 |  |  | 100 |  |
| 7-22 | 27 |  |  |  | 2.5 |  |  | 100 |  |
| 7-23 | 28 |  |  |  | 2.5 |  |  | 100 |  |
| 7-24 | 29 |  |  |  | 2.5 |  |  | 100 |  |
| 7-25 | 30 |  |  |  | 2.5 |  |  | 100 |  |
| 7-26 | 31 |  |  |  | 2.5 |  |  | 100 |  |
| 7-27 | 32 |  |  |  |  |  |  | 100 |  |
| 7-28 | 33 |  |  |  |  |  |  | 100 |  |
| 7-29 | 34 |  |  |  |  |  |  | 100 |  |
| 7-30 | 35 |  |  |  |  |  |  | 100 | Blast cell: 2.5%,  MRD: 15.1% |
| 8-26-2020 |  |  |  |  |  |  |  | 100 | Blast cell: 1%,  MRD: 5.2*10E-3。qPCR: TERF2-PDGFRB 0.99% |

CTX:cyclophosphamide, IDA:idarubicin, DEX:dexamethasone, Ara-c:Cytarabine, VP-16:etoposide
